# Load‐To‐Failure of Cantilevered Provisional Implant‐Supported Prostheses: Conventional Versus 3D‐Printed Resins

**DOI:** 10.1111/jerd.70164

**Published:** 2026-04-20

**Authors:** Térsia Cristina Silva Macedo, Carolina Neves Tannous Dib, Maribi Isomar Terán Lozada, João Pedro Alves Silva, Karla Zancopé

**Affiliations:** ^1^ Uberlândia Uberlândia Minas Gerais Brazil; ^2^ Department of Occlusion, Fixed Prosthesis and Dental Materials, School of Dentistry Federal University of Uberlândia Minas Gerais Brazil; ^3^ Department of Restorative Dentistry and Dental Materials, School of Dentistry Federal University of Uberlândia Uberlândia Minas Gerais Brazil

**Keywords:** dental materials, edentulous, flexural strength, implant‐supported dental prosthesis, temporary denture

## Abstract

**Objective:**

Cantilever extensions in full‐arch implant‐supported provisional prostheses represent a clinical challenge frequently associated with biomechanical complications. This study aimed to evaluate the load‐to‐failure and deflection of acrylic and 3D‐printed resins used in the cantilever region of implant‐supported provisional prostheses fabricated over mini‐abutment cylinders.

**Materials and Methods:**

Forty standardized specimens with 15‐mm cantilever were allocated into four groups (*n* = 10): auto‐polymerized resin (RA), high‐impact thermo‐polymerized resin (RD), 3D‐printed resin (RI) and thermo‐polymerized denture resin reinforced with metallic infrastructure (RT, control). Load‐to‐failure and displacement were measured using a universal testing machine and deflectometer (Instron). Data were analyzed using one‐way ANOVA and Tukey's post hoc test (*α* = 0.05).

**Results:**

Significant differences were observed among all materials (*p* < 0.001). The control group showed the highest load‐to‐failure (553 N), followed by RI (506 N), RD (440 N), and RA (396 N). The 3D‐printed resin group exhibited the lowest deflection value (2.33 mm) and the high‐impact resin the greatest (5.07 mm).

**Conclusion:**

Material selection critically influences the biomechanical stability of cantilevered provisional implant prostheses. The high‐impact resin showed higher degree of flexibility; and the RI group greater load bearing capacity, time efficiency, and predictability, therefore both are suitable alternatives for full‐arch temporary implant‐supported rehabilitations.

## 1 Introduction

Edentulism is an oral condition that significantly impacts the physical and mental health of individuals who have completely lost their teeth [1]. Globally, the number of edentulous people is expected to reach nearly 661 million by 2050 [2]. To treat this condition, complete‐arch implant‐supported fixed dental prostheses (FDPs) are widely recommended [3, 4, 5], since it is considered a reliable treatment option due to their high success rates and predictable clinical outcomes [5, 6, 7].

However, dental implants cannot always be placed in the ideal position, due to the risk of damaging nerves and blood vessels. To address this clinical challenge, FDPs with distal cantilever are often planned. The cantilever represents a significant point of vulnerability in the prosthesis, as it is a suspended structure in the posterior area, thus entails mechanical limitations, particularly in temporary rehabilitations [8, 9]. Occlusal loads can generate high stress and strain in this area, potentially leading to fracture of the distal part of the prosthesis [5, 10].

Determining the bite force is a crucial parameter in assessing the efficacy of dental prostheses [11]. Occlusal overload may contribute to implant or prosthesis failure, as the applied force may surpass the load‐bearing capacity of the selected restorative material [10, 12]. In multiple implant‐supported prostheses the fractures tend to occur mainly at the distal implant level [13]. Mean values for the maximum bite force pattern range from approximately 547 N to 631 N, according to recent studies [14, 15].

The placement of a temporary implant‐supported prosthesis is an intermediary treatment intended to protect periodontal tissues, promote guided tissue healing and properly manage emergence profiles [16]. Temporary prostheses must withstand occlusal loads throughout the osseointegration process, which can last from 3 to 6 months [17, 18]. Therefore, it must be manufactured using materials that resist the tissue healing period and the osseointegration process [18]. Fractures in the cantilevered region may occur due to stress and strain during chewing or parafunctional habits, causing failure of the prosthesis during the healing phase [19]. Frequent repairs are often necessary, and conventionally auto‐polymerized resins are the most widely used material due to their esthetics, easy manipulation, and low cost [20]. However, this material has relatively low mechanical resistance, which can lead to prosthesis fracture [19].

Additionally, given the growing adoption of digital dentistry workflows and its well‐established advantages reported in the literature, such as reduced treatment time, greater predictability, and enhanced patient satisfaction [21, 22, 23], it could be an option for implant‐supported temporary rehabilitation. Computer‐assisted manufacturing techniques, especially 3D printing, presents satisfactory accuracy, reduced operator dependency, and shorter production times compared with conventional analog workflows, potentially minimizing the likelihood of errors [24]. The use of ceramic‐filled 3D‐printed requires appropriate cementation and post‐curing protocols for their clinical indication [25, 26], when these conditions are properly followed, it could be a viable temporary material for implant‐supported complete dentures [27]. While the literature reports an increasing number of successful cases involving removable complete dentures and full‐coverage crowns fabricated with printed resins [23, 24], further research is still required regarding FDP's.

It is crucial to understand the mechanical behavior of dental materials through laboratory studies, such as their load‐to‐failure and deflection, as these properties indicate their safety for clinical application [28]. To the best of the authors' knowledge, the biomechanical behavior of temporary implant‐supported prostheses made from different materials has not been fully elucidated. Therefore, the aim of the present study was to evaluate the load‐to‐failure and deflection in the cantilever region of provisional implant‐supported prostheses fabricated on conventional acrylic resins and 3D‐printed resin, using thermo‐polymerized resin reinforced with metallic infrastructure as the control group.

The hypothesis was that the mechanical behavior of the cantilevers of provisional implant‐supported prostheses would differ among the manufacturing material.

## 2 Materials and Methods

This study was an in vitro laboratory test. The experimental unit consisted of provisional implant‐supported prostheses with a distal cantilever (*n* = 10 per group), divided into four groups (Table 1). The sample size was established according to previous studies that indicated the minimum number of specimens required to achieve reliable and robust statistical analysis for this methodological design [29].

**TABLE 1:** Groups classified as the fabrication resin of the experimental units.

Group	Material	Commercial name
RA	Conventionally auto‐polymerized resin	VIPI flash—self‐curing acrylic resin
RD	High‐impact thermo‐polymerized resin	Diamond D thermocuring acrylic resin, keystone
RT	Conventionally thermo‐polymerized denture resin with metallic infrastructure	VIPI cril plus (VIPI) with nickel chromium (Wironia light, BEGO)
RI	3D‐printed resin	ONX tough 2, bleach (SprintRay)

Abbreviations: 3D‐printed resin (RI group); conventionally auto‐polymerized resin (RA group); conventionally thermo‐polymerized denture resin with metallic structure (RT group); high resistance thermo‐polymerized resin (RD group).

### 2.1 Obtention of Support

**FIGURE 1:**
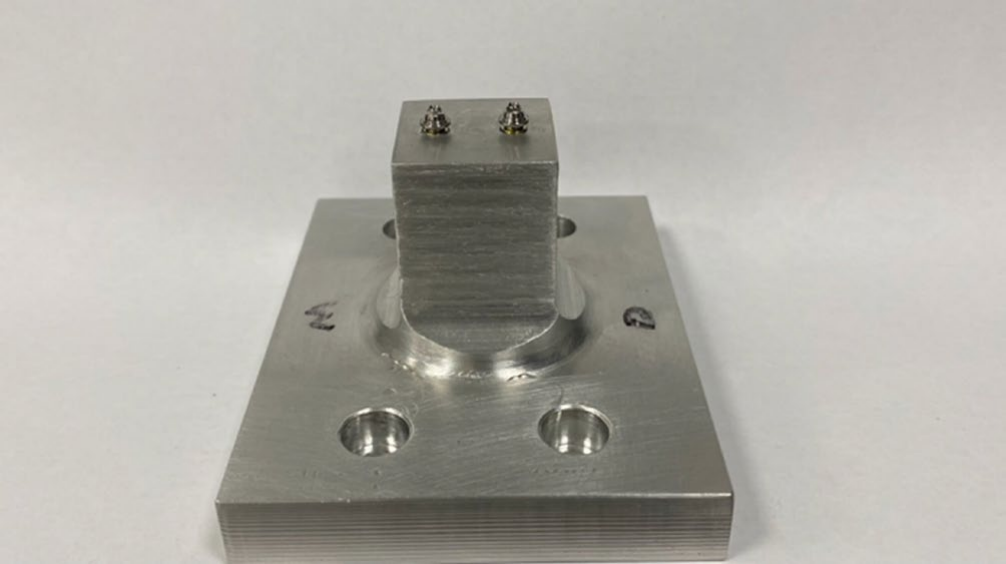
Metal base with analogues and mini abutments in position.

A metallic support was designed for a perfect fit in the universal testing machine (ElectroPuls E3000, Instron, Norwood, Massachusetts, United States of America), allowing for the performance of all tests. The support was made from aluminum, with dimensions of 90 mm long, 70 mm wide and 12 mm high. In the center of the base, a square structure (25 mm wide, 25 mm long, and 35 mm high) was machined (Figure 1). Two perforations were made to place 2 dental implant analogs (GM 4.1, Neodent, Curitiba, Paraná, Brazil) positioned 1 cm equidistantly. Two mini abutments (1.5, Neodent Curitiba, Paraná, Brazil) were placed in their respective analogs, torqued according to the manufacturer's recommendation. Transfers were then screwed over the abutments, forming an index to transfer the exact position of the analogs.

### 2.2 RT Group (Conventional Resin With Metallic Infrastructure)

**FIGURE 2:**
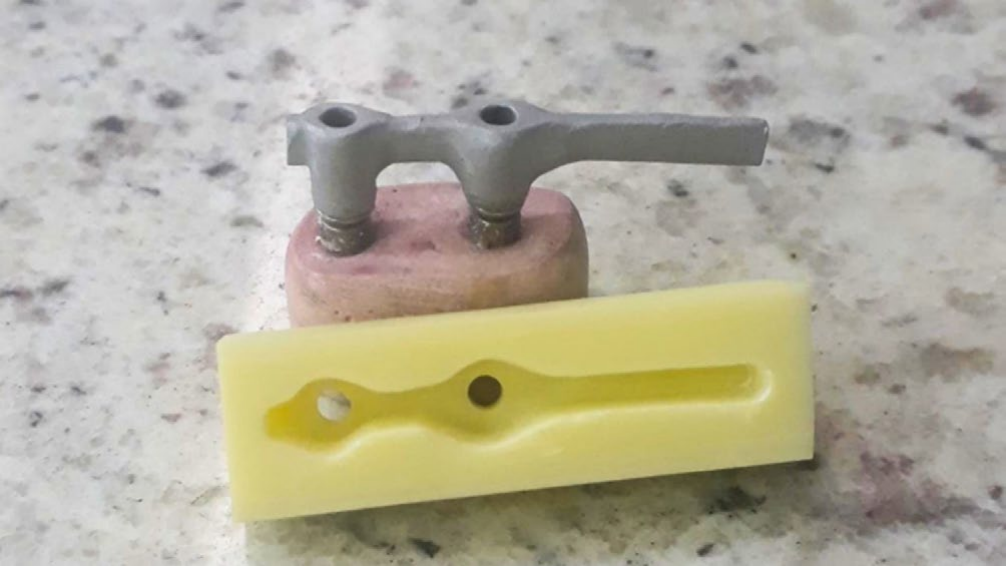
Metallic infrastructure cast finished in nickel chrome and printed resin bar that will be used to pour the study resins.

With the index screwed onto the metal base, the positive control group was obtained. The mini abutments position was scanned (InEos X5, Dentsply Sirona, Charlotte, North Carolina, United States of America), and the metal bar was designed using CAD software (EXOCAD, Align Technology, Darmstadt, Hesse, Germany). Each bar had a 35 mm length, a 4 mm thickness, and a 15 mm cantilever. Another rectangular bar was designed (EXOCAD, Align Technology), with 10 mm wide and 8 mm high. From the CAD drawings, 10 bars for the infrastructure and the rectangular bases were 3D printed (Anycubic, Shenzhen, Guangdong, China). The bars followed the conventional casting process, including investment, inclusion, and casting via the lost wax technique with a nickel‐chromium metal alloy (Wironia light, BEGO, Bremen, Bremen, Germany) (Figure 2).

The cast bars were positioned in the resin pattern with the final shape of the resin base, surrounding the metal bar. The inclusion process in the flask was performed conventionally (type IV dental stone, DentMix Type IV Special Stone, Asfer, São Caetano do Sul, São Paulo, Brazil). Six samples were positioned inside the flask at a time. The thermo‐polymerized resin was placed over the space left by the printed resin bars, and polymerization was performed using a microwave oven (1200 to 1400 W; initial stage: 20 min with 10/20% power; and final stage: + 5 min with 30/40% power). Finishing and polishing were carried out on all samples.

### 2.3 Resin Groups Without Metallic Infrastructure (RA, RD, and RI Groups)

The titanium cylinders (GM mini abutments 4.1, Neodent) were captured over the implant analogs (GM analog implant 4.1, Neodent) and included in the flask, as described above. The polymerization cycles used for each resin were as follows:

– RD Group: High‐impact thermo‐polymerized resin (Diamond D, Keystone, Gibbstown, New Jersey, United States of America) was used in a ratio of 10 mL of monomer to 21 g of powder. Polymerization was performed in a microwave oven for 4 min at 700 W, followed by finishing and polishing of all samples.
– RA Group: Conventionally auto‐polymerized resins (Self‐curing acrylic resin, VIPI, Pirassununga, São Paulo, Brazil). Two portions of powder to 1 portion of liquid (14 g of powder to 7 mL of liquid) were carefully homogenized. For polymerization, the resin was taken to a polymerizer with water, under 20 pounds of compressed air, and allowed complete polymerization for 20 min before opening the flask, removing the parts, and finishing and polishing all samples.
– RI Group: 3D‐printed resin containing ceramic nanoparticles (ONX Tough 2, Bleach, SprintRay, Los Angeles, California, United States of America). The resin bars were designed and printed using a 3D printer (SprintRay Pro S55 printer, SprintRay), and then washed (SprintRay Pro Wash 2, SprintRay) and post‐cured (Nano Cure 2 chamber, SprintRay), following the manufacturer's instruction. The cylinder abutments were cemented to the resin bar using the same 3D‐printing resin, initially it was light‐cured for 40 s on the buccal and lingual surfaces, followed by post‐curing in a specific curing unit for 40 min.

### 2.4 Load‐To‐Failure Test

The metal base assembly and each structure were tested using the universal testing machine (ElectroPuls E3000, Instron). A rounded piston was positioned at 5 mm from the end of the specimen, simulating biting over the cantilever. An axial and progressive load was applied until fracture, and the maximum load to failure (N) and displacement (mm) were obtained. A deflectometer (E‐Series Deflectometers, Instron) was positioned in the lower part of the cantilever to measure the displacement at the moment of failure.

### 2.5 Statistical Analysis

Data were first analyzed for normal distribution (Shapiro–Wilk test) and homoscedasticity (Levene's test). Subsequently, a one‐way analysis of variance (ANOVA) followed by Tukey's post hoc tests was used to compare the load‐to‐failure (N) and deflection (mm) data. All tests used a significance level of *α* = 0.05, and all analyses were carried out with statistical software (JAMOVI, The Jamovi Project Version 2.4, Sydney, New South Wales, Australia).

## 3 Results

The means and standard deviations of the maximum load to failure (N) and the deflection (mm) at the moment of failure are shown in Table 2. A significant difference was found between the auto‐polymerized resin (RA), when compared to high‐impact thermo‐polymerized resin (RD), 3D‐printed resin containing ceramic nanoparticles (RI), and conventionally thermo‐polymerized denture resin (RT) groups (*p* < 0.001) (Table 2).

**TABLE 2:** Measured variables.

Group	Load‐to‐failure (N)	Deflection (mm)
RA	396 A ± 58	2.37 ± 0.85
RD	440 B ± 28	5.07 ± 1.04
RT	553 B ± 67	3.93 ± 1.33
RI	506 B ± 108	2.33 ± 0.47

*Note:* Descriptive analysis of data of load‐to‐failure (N) and deflection (mm) at the maximum bending moment of each tested group. Different letters (A, B) indicate statistically significant differences between groups.

The results for Bending Displacement at maximum force and Deflection at maximum force tests demonstrated that the high‐impact acrylic resin (RD) has a significantly greater deformation before fracture occurs compared to the other three resins.

**FIGURE 3:**
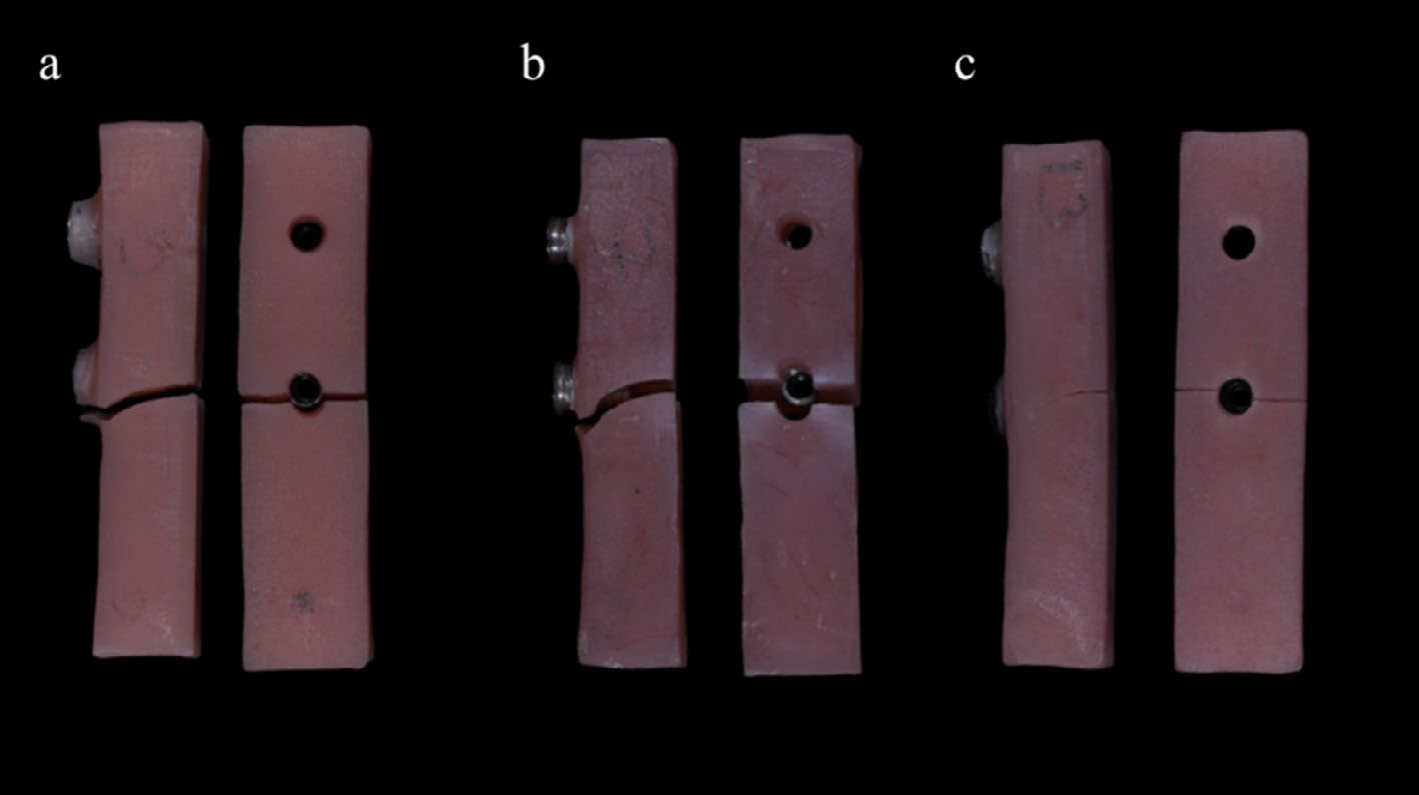
Example of how fractures of the resin samples looked after tests: (a) Conventionally self‐polymerized resins (RA), (b) High resistance Thermo‐polymerized resin (RD); and (c) Conventionally Thermo‐polymerized denture resin (RT).

**FIGURE 4:**
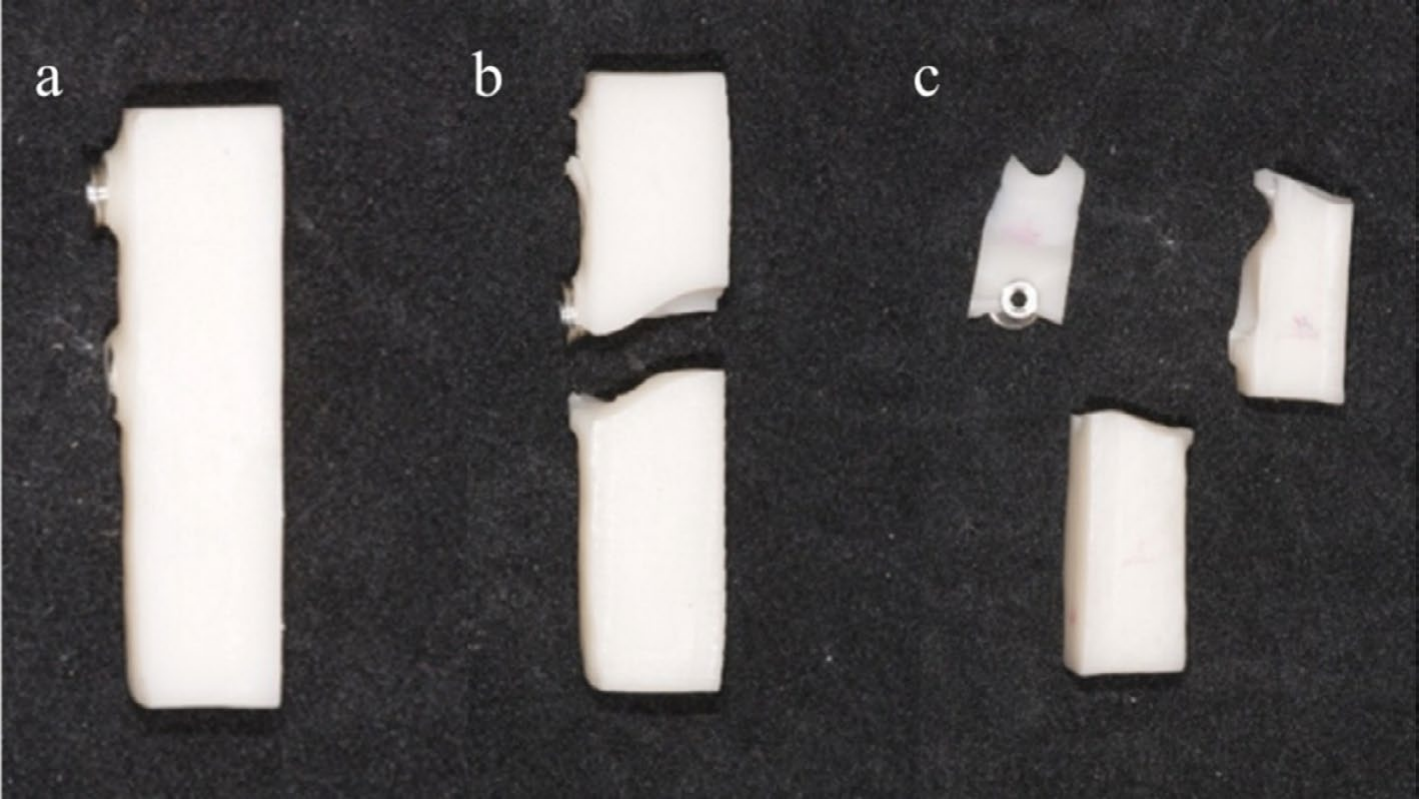
Examples of the fracture patterns observed in RI group: (a) Mesial cylinder debonding, (b) Linear fracture at the distal cylinder abutment, and (c) Fragmented fracture.

All samples were photographed, and the fracture mode was analyzed. In groups RA, RD, and RT, all fractures occurred at the distal cylinder abutment (Figure 3). However, the RI group exhibited different deformation patterns, which the authors classified as: mesial cylinder debonding (a)–three samples; Linear fracture at the distal cylinder abutment (b)–five samples; and Fragmented fracture (c)–four samples (Figure 4).

## 4 Discussion

The hypothesis of the present study was accepted, as significant differences were detected in the mechanical behavior of the provisional implant‐supported prostheses with distal cantilevers, depending on the material used. In all groups, failures predominantly occurred on the distal abutment cylinders. This outcome aligns with expectations and is a common phenomenon in failures due to the absence of posterior reinforcement [5, 10]. In full‐arch implant‐supported prostheses fabricated with different combinations of mesostructure and veneering materials fractures tended to occur mainly at the distal implant level [13], which is consistent with the findings of the present study. This type of fracture occurs because the point of force concentration is distant from the distal component, as the fracture occurs through the slender lateral walls of the screw capture hole in the furthest part of the distal component [8]. In the absence of a metal bar to reinforce the prosthesis, repair is unfeasible, considering that the resin becomes entirely detached. In a hypothetical scenario where the dentist chooses an implant‐supported provisional prosthesis made of autopolymerizing acrylic resin (RA), the material may not be appropriate, since, according to the findings of this study, the average maximum force supported by the RA group is 396 N, and the mean bite force shown in recent studies stands at 547 N [14]; therefore, this prosthesis exhibits a higher risk of fracture.

The RI group exhibited different deformation patterns. Several factors may explain this variability. Regarding type “a” (mesial cylinder debonding), failure likely occurred in the cementation between the mini‐abutment cylinders and the resin bars. This phenomenon may have resulted either from the cementation protocol employed [25] or from issues related to the polymerization of the 3D printing resin used as a bonding agent, whether during the light‐curing step or the post‐curing process in the specific curing chamber [26]. Concerning the fracture classified as type “c” (fragmented fracture), a possible explanation lies in the distribution and condition of the ceramic nanoparticles dispersed within the resin, which influence the local stiffness and the initiation and propagation of cracks. Although ceramic nanoparticles increase the overall strength of the resin, when agglomerated they create concentration of local stress that may lead to irregular fracture patterns [27], such as those observed in this experiment. Post‐processing procedures may also affect the stiffness of the specimens, as they directly influence the mechanical strength of the material and can therefore modify the resulting fracture pattern [26]. Hence, it highlights the importance of proper calibration of the 3D printing resin and the post‐curing process to ensure that both the prosthetic framework and the chosen material fulfill their expected functional performance.

The use of temporary prostheses manufactured with self‐polymerizing acrylic resin is common due to its advantages, such as esthetics, ease of manipulation, and low cost [20]. However, accordingly to the worst result of the force supported among the tested groups showed in this study, this material is not recommended for cases that require high mechanical strength in virtue of its relatively low mechanical resistance.

**FIGURE 5:**
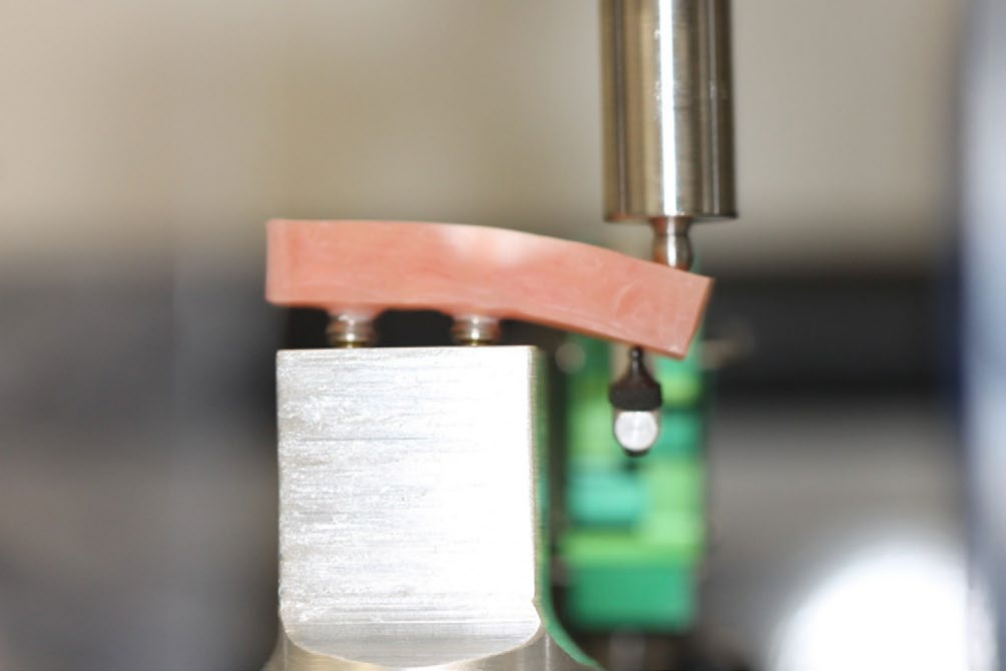
Sample of high‐resistance resin close to the fracture, image shows how much it can deflect, and how it turns white in the region where the force is greatest.

According to the results obtained in this study, the use of high‐impact resins is preferable than self‐polymerizing acrylic resin, especially when the patient's situation demands a longer period of osseointegration, which means the provisional prostheses will need to remain in function for a longer period [17]. This resin supports higher force than the acrylic resin and has the highest deflection among all the tested groups. During tests, the RD group showed areas with different colors, indicating the possible overload site in the resin bar; this area showed whitening before the fracture (Figure 5). Thus, it is expected that the dentist will be able to observe, during follow‐up appointments, whether the distribution of occlusal forces is appropriate or if there are any overloading points and adjustments that need to be made.

When discussing the fabrication time of a prosthesis, particularly in full‐arch rehabilitations, time is a crucial factor in determining if a given manufacturing material is worth adopting. As the number of edentulous patients continues to rise, the clinical demand, together with that of dental technicians, increases accordingly [2, 5]. In this context, the use of 3D‐printed resins reinforced with ceramic fillers provides professionals with both accuracy and efficiency during the laboratory procedures and, consequently, throughout the overall prosthetic treatment of edentulous patients. This combination represents an excellent cost‐effective option for the fabrication of implant‐supported provisional complete dentures [21, 22].

From this perspective, when a prosthesis undergoes a non‐repairable fracture, the re‐fabrication process becomes much simpler with a fully digital additive manufacturing workflow. Since the original digital design of the prosthesis can be reprinted directly, additional clinical appointments for new impressions or implant scanning are unnecessary. Furthermore, this approach allows rapid reprinting and delivery of the final prosthesis ready for intraoral installation.

This paper highlights the need for in vivo studies to better understand the behavior of these materials in implant‐supported provisional prostheses with cantilevers in humans, thereby expanding knowledge about their indications and limitations according to patient singularities. For this reason, further in vivo studies on this topic are warranted.

## 5 Conclusions

Within the limitations of this study, it was concluded that:

1. The material used in provisional implant‐supported prostheses with cantilevers significantly affect their load‐to‐failure and deflection.
2. The reinforced thermo‐polymerized resin group demonstrated the highest load‐to‐failure, while the acrylic resin group showed the lowest mechanical behavior.
3. The high‐impact thermo‐polymerized resin group showed the greatest deflection before fracture, indicating higher degree of flexibility, therefore represents a suitable alternative for this purpose.
4. The 3D‐printed resin with ceramic nanoparticles is considered a material option due to its high load‐to‐failure, time efficiency and predictability.

## Funding

This work was supported by Coordenação de Aperfeiçoamento de Pessoal de Nível Superior, 001, Conselho Nacional de Desenvolvimento Científico e Tecnológico, 406840/2022‐9, Fundação de Amparo à Pesquisa do Estado de Minas Gerais, RED‐00204‐23.

## Conflicts of Interest

The authors declare no conflicts of interest.

## Data Availability

The data that support the findings of this study are available from the corresponding author upon reasonable request.

## References

[1] R. C. Ferreira , A. M. D. Vargas , R. N. V. de Moura , et al., “Caries and Edentulism Trends Among Brazilian Older Adults: A Comparative Analysis of 2003, 2010, and 2023 Surveys,” Brazilian Oral Research 39, no. suppl 1 (2025): e050, 10.1590/1807-3107bor-2025.vol39.0050. 40396854 PMC12096856

[2] G. G. Nascimento , S. Alves‐Costa , and M. Romandini , “Burden of Severe Periodontitis and Edentulism in 2021, With Projections up to 2050: The Global Burden of Disease 2021 Study,” Journal of Periodontal Research 59, no. 5 (2024): 823–867, 10.1111/jre.13337. 39192495

[3] U. Soboleva and I. Rogovska , “Edentulous Patient Satisfaction With Conventional Complete Dentures,” Medicina (Kaunas, Lithuania) 58, no. 3 (2022): 344, 10.3390/medicina58030344. 35334520 PMC8953744

[4] M. Novais , A. S. Silva , J. Mendes , P. Barreiros , C. Aroso , and J. M. Mendes , “Fracture Resistance of CAD/CAM Implant‐Supported 3Y‐TZP‐Zirconia Cantilevers: An In Vitro Study,” Materials 15, no. 19 (2022): 6638, 10.3390/ma15196638. 36233980 PMC9571496

[5] Y. Tang , H. Yu , J. Wang , and L. Qiu , “Implant Survival and Complication Prevalence in Complete‐Arch Implant‐Supported Fixed Dental Prostheses: A Retrospective Study With a Mean Follow‐Up of 5 Years,” International Journal of Oral & Maxillofacial Implants 38, no. 1 (2023): 84–93, 10.11607/jomi.9808. 37099585

[6] K. M. Alzahrani , “Implant Bio‐Mechanics for Successful Implant Therapy: A Systematic Review,” Journal of International Society of Preventive and Community Dentistry 10, no. 6 (2020): 700–714, 10.4103/jispcd.JISPCD_138_20. 33437702 PMC7791586

[7] S. Rues , S. Kappel , D. Ruckes , P. Rammelsberg , and A. Zenthöfer , “Resistance to Fracture in Fixed Dental Prostheses Over Cemented and Screw‐Retained Implant‐Supported Zirconia Cantilevers in the Anterior Region: An In Vitro Study,” International Journal of Oral & Maxillofacial Implants 35, no. 3 (2020): 521–529, 10.11607/jomi.7899. 32406648

[8] A. Roccuzzo , R. Fanti , L. Mancini , et al., “Implant‐Supported Fixed Dental Prostheses With Cantilever Extensions: State of the Art and Future Perspectives,” International Journal of Oral Implantology 16, no. 1 (2023): 13–28. 36861678

[9] A. Aboelfadl , L. Keilig , K. Ebeid , et al., “Biomechanical Behavior of Implant Retained Prostheses in the Posterior Maxilla Using Different Materials: A Finite Element Study,” BMC Oral Health 24, no. 1 (2024): 455, 10.1186/s12903-024-04142-8. 38622680 PMC11020654

[10] Y. Kondo , K. Sakai , H. Minakuchi , T. Horimai , T. Kuboki , and JSOI Clinical Guideline Working Group collaborators , “Implant‐Supported Fixed Prostheses With Cantilever: A Systematic Review and Meta‐Analysis,” International Journal of Implant Dentistry 10, no. 1 (2024): 57, 10.1186/s40729-024-00573-8. 39570465 PMC11582258

[11] S. Levartovsky , G. Peleg , S. Matalon , I. Tsesis , and E. Rosen , “Maximal Bite Force Measured via Digital Bite Force Transducer in Subjects With or Without Dental Implants—A Pilot Study,” Applied Sciences 12 (2022): 1544, 10.3390/app12031544.

[12] M. Manfredini , P. P. Poli , L. Giboli , M. Beretta , C. Maiorana , and M. Pellegrini , “Clinical Factors on Dental Implant Fractures: A Systematic Review,” Dentistry Journal 12, no. 7 (2024): 200, 10.3390/dj12070200. 39056987 PMC11276356

[13] E. Haroyan‐Darbinyan , M. Romeo‐Rubio , J. D. Río‐Highsmith , C. D. Lynch , and R. Castillo‐Oyagüe , “Fracture Resistance of Cantilevered Full‐Arch Implant‐Supported Hybrid Prostheses With Carbon Fiber Frameworks After Thermal Cycling,” Journal of Dentistry 116 (2022): 103902, 10.1016/j.jdent.2021.103902. 34822914

[14] I. Nitschke , C. Moede , A. Koenig , B. A. J. Sobotta , W. Hopfenmüller , and J. Jockusch , “An Evaluation of Reference Bite Force Values: Investigating the Relationship Between Dental Prosthetic Restoration and Bite Force in a Cross‐Sectional Study,” Journal of Clinical Medicine 14, no. 8 (2025): 2723, 10.3390/jcm14082723. 40283551 PMC12027650

[15] S. R. Patil , G. Maragathavalli , D. N. S. V. Ramesh , G. S. Naidu , M. K. Alam , and I. A. AlZoubi , “The Reliability of a New Device for Measuring the Maximum Bite Force,” BioMed Research International 2022 (2022): 3272958, 10.1155/2022/3272958. 35071592 PMC8776449

[16] V. J. J. Donker , G. M. Raghoebar , A. Vissink , and H. J. A. Meijer , “Digital Workflow for Immediate Implant Placement and Chairside Provisionalization in the Esthetic Zone,” Case Reports in Dentistry 2022, no. 1 (2022): 5114332, 10.1155/2022/5114332. 35527725 PMC9076344

[17] C. Pandey , D. Rokaya , and B. P. Bhattarai , “Contemporary Concepts in Osseointegration of Dental Implants: A Review,” BioMed Research International 2022 (2022): 6170452, 10.1155/2022/6170452. 35747499 PMC9213185

[18] S. L. Barua , T. S. Poduval , S. Rani , N. Jain , and S. Thakur , “Stress Distribution in Bone Around an Implant‐Supported Three‐Unit Fixed Dental Prosthesis Using Two Different Computer‐Aided Designing/Computer‐Aided Milling Provisional Crown Materials: Milled Polymethylmethacrylate and Milled Polyetheretherketone ‐ A Finite Element Analysis,” Dental Research Journal 20 (2023): 33. 37180686 PMC10166750

[19] K. Angelara , M. Bratos , and J. A. Sorensen , “Comparison of Strength of Milled and Conventionally Processed PMMA Complete‐Arch Implant‐Supported Immediate Interim Fixed Dental Prostheses,” Journal of Prosthetic Dentistry 129, no. 1 (2023): 221–227, 10.1016/j.prosdent.2021.04.025. 34158174

[20] H. Yu , J. Yao , Z. Du , J. Guo , and W. Lei , “Comparative Evaluation of Mechanical Properties and Color Stability of Dental Resin Composites for Chairside Provisional Restorations,” Polymers 16, no. 14 (2024): 2089, 10.3390/polym16142089. 39065406 PMC11280800

[21] D. H. Ballard , P. Mills , R. Duszak, Jr. , J. A. Weisman , F. J. Rybicki , and P. K. Woodard , “Medical 3D Printing Cost‐Savings in Orthopedic and Maxillofacial Surgery: Cost Analysis of Operating Room Time Saved With 3D Printed Anatomic Models and Surgical Guides,” Academic Radiology 27, no. 8 (2020): 1103–1113, 10.1016/j.acra.2019.08.011. 31542197 PMC7078060

[22] Y. Tian , C. Chen , X. Xu , et al., “A Review of 3D Printing in Dentistry: Technologies, Affecting Factors, and Applications,” Scanning 2021 (2021): 9950131, 10.1155/2021/9950131. 34367410 PMC8313360

[23] C. Auduc , T. Douillard , E. Nicolas , and N. El Osta , “Fully Digital Workflow in Full‐Arch Implant Rehabilitation: A Descriptive Methodological Review,” PRO 7 (2025): 85, 10.3390/prosthesis7040085.

[24] M. Popescu , V. S. Perieanu , M. Burlibașa , et al., “Comparative Cost‐Effectiveness of Resin 3D Printing Protocols in Dental Prosthodontics: A Systematic Review,” PRO 7 (2025): 78, 10.3390/prosthesis7040078.

[25] T. Filokyprou , M. J. Kesterke , X. Liu , S. H. Cho , and M. Revilla‐León , “Effect of Different Surface Treatments on the Retention Force of Additively Manufactured Interim Implant‐Supported Crowns,” Journal of Prosthodontics 33, no. 9 (2024): 899–907, 10.1111/jopr.13783. 37823323

[26] S. Kirby , I. Pesun , A. Nowakowski , and R. França , “Effect of Different Post‐Curing Methods on the Degree of Conversion of 3D‐Printed Resin for Models in Dentistry,” Polymers 16, no. 4 (2024): 549, 10.3390/polym16040549. 38399926 PMC10892052

[27] A. Alshamrani , A. Alhotan , E. Kelly , and A. Ellakwa , “Mechanical and Biocompatibility Properties of 3D‐Printed Dental Resin Reinforced With Glass Silica and Zirconia Nanoparticles: In Vitro Study,” Polymers 15, no. 11 (2023): 2523, 10.3390/polym15112523. 37299322 PMC10255304

[28] L. Bein , A. Rauch , M. Schmidt , and M. Rosentritt , “In Vitro Fatigue and Fracture Testing of Temporary Materials From Different Manufacturing Processes in Implant‐Supported Anterior Crowns,” Clinical Oral Investigations 27, no. 8 (2023): 4215–4224, 10.1007/s00784-023-05038-7. 37133699

[29] E. Zancopé , K. Zancopé , A. V. Carvalho Pinto , et al., “Influence of the Prosthetic Interface and Manufactured Metal on the Mechanical Behavior of Two Extra‐Narrow Implants,” International Journal of Oral & Maxillofacial Implants 41, no. 1 (2026): 69–76, 10.11607/jomi.10960. 40553618

